# Cost-effectiveness analysis of carbetocin for prevention of postpartum hemorrhage in a low-burden high-resource city of China

**DOI:** 10.1371/journal.pone.0279130

**Published:** 2022-12-15

**Authors:** Joyce H. S. You, Tak-yeung Leung

**Affiliations:** 1 School of Pharmacy, Faculty of Medicine, The Chinese University of Hong Kong, Hong Kong SAR, China; 2 Department of Obstetrics and Gynaecology, Faculty of Medicine, The Chinese University of Hong Kong, Hong Kong SAR, China; Istituto Superiore di Sanità and St. Camillus International University of Health Sciences, ITALY

## Abstract

**Background:**

Postpartum hemorrhage (PPH) is a major cause of maternal morbidity, and oxytocin is the first-line uterotonic agent for PPH prevention. Clinical findings have reported carbetocin to reduce PPH risk without increasing risk of important side effects. Hong Kong is a low PPH burden and high-resource city in China. We aimed to examine the cost-effectiveness of PPH prevention with carbetocin from the perspective of Hong Kong public healthcare provider.

**Methods:**

A decision-analytic model was developed to simulate clinical and economic outcomes of carbetocin and oxytocin for PPH prevention in a hypothetical cohort of women at the third stage of labor following vaginal birth or Caesarean section (C-section). The model inputs were retrieved from literature and public data. Base-case analysis and sensitivity analysis were performed. The model time horizon was the postpartum hospitalization period. Primary model outcomes included PPH-related direct medical cost, PPH, hysterectomy, maternal death, and quality-adjusted life-year (QALY) loss.

**Results:**

In base-case analysis, carbetocin (versus oxytocin) reduced PPH-related cost (by USD29 per birth), PPH ≥500 mL and ≥1,500 mL (by 13.7 and 1.9 per 1,000 births), hysterectomy (by 0.15 per 1,000 births), maternal death (by 0.02 per 1,000 births), and saved 0.00059 QALY per birth. Relative risk of PPH ≥500 mL with carbetocin versus oxytocin, and proportion of child births by C-section were two influential parameters identified in deterministic sensitivity analysis. In probabilistic sensitivity analysis, carbetocin was accepted as cost-effective in >99.7% of the 10,000 Monte Carlo simulations at a willingness-to-pay threshold of zero USD/QALY.

**Conclusion:**

PPH prevention with carbetocin appeared to reduce major unfavorable outcomes, and save cost and QALYs.

## Introduction

Postpartum hemorrhage (PPH) is a major cause of maternal morbidity including serious events such as coagulopathy, acute respiratory failure, and renal failure associated with hypovolemic shock and resuscitation [1, 2]. PPH is frequently defined as blood loss of ≥500 mL from the genital tract within 24 hours of childbirth [3]. The prevalence of PPH has been more prominent in the developing regions, yet it is rising and ranges 3%-8% in the developed countries [3–5]. In Hong Kong, a high-resource city of China, the PPH prevalence was reported to increase from 4.8% to 6.2% over 2013–2017. Despite the relative low prevalence of PPH, the disease burden associated with PPH-related management is high in Hong Kong, including first-line uterotonic drug treatment, second-line therapies (balloon tamponade, compression sutures, and artery embolisation or surgical ligation), and hysterectomy [6].

The use of uterotonic agent is the recommended prophylaxis for prevention of PPH, and oxytocin is the first-line uterotonic agent [3]. Carbetocin is a synthetic analogue of oxytocin, with enhanced half-life, duration and stability [7]. A recent meta-analysis of 196 clinical trials (n = 135,559) reported by the Cochrane Review had showed that the use of carbetocin was associated with reduced risk of PPH ≤500 mL (RR 0.72 (95% CI 0.56 to 0.93)) when compared to oxytocin, without increasing the risk of important side effects (such as vomiting and fever) [8]. Findings of prior studies have suggested carbetocin to reduce total direct medical cost and PPH events, and gained higher quality-adjusted life-years (QALYs) (comparing to oxytocin) in the UK [9–11]. The Hospital Authority is the sole public healthcare provider in Hong Kong, and manages 43 public hospitals/institutes and over 120 outpatient clinics. Obstetrics inpatient service is provided by eight of the 43 public hospitals/institutes, each with bed number ranges 1,100–1,900 [12]. Despite the reduced risk of PPH associated with carbetocin, the cost of carbetocin (USD25.6 per single dose 100mcg; USD1 = HKD7.8) is much higher than the drug cost of oxytocin (USD1.03 per 10 IU; dose range 10–40 IU) in the Hospital Authority of Hong Kong [6]. To inform clinicians and healthcare administrators on decision-making of public resource allocation, the present study aimed to examine the cost-effectiveness of carbetocin for the prevention of PPH from the perspective of Hong Kong public healthcare provider.

## Methods

### Model structure

**Fig 1:**
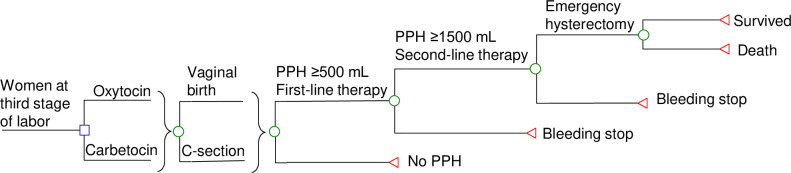
Simplified decision-analytical model for PPH prevention with carbetocin versus oxytocin. PPH: Postpartum hemorrhage.

A decision-analytic model (Fig 1) was developed to simulate the clinical and economic outcomes of two uterotonic agents (carbetocin and oxytocin) for PPH prevention in a hypothetical cohort of women at the third stage of labor following vaginal birth or Caesarean section (C-section). Decision tree is a form of decision-analytical model in which hypothetical patients proceed through health states over time based on probability inputs of the model. The model time horizon was the postpartum hospitalization period. Primary model outcomes included PPH-related direct medical cost, PPH, emergency hysterectomy, death, and QALY loss.

At the entry of model, a hypothetical patient (delivered child by vaginal birth or C-section) received PPH prevention with oxytocin 10 IU (intravenous bolus) or carbetocin 100 mcg (intravenous infusion). In both study groups, the hypothetical patient might experience postpartum blood loss ≥500 mL. If blood loss was ≥500 mL, first-line treatment would be initiated with uterotonic agents (oxytocin-ergometrine infusion, carboprost), and stepped up (when bleeding continued to ≥1,500 mL) to second-line therapies (balloon tamponade, compression sutures of uterine, and artery embolisation or surgical ligation) and emergency hysterectomy [13].

### Clinical inputs

The parameters used in the present model are shown in Table 1. A literature search on Medline over the period 2000–2022 was performed using keywords such as “postpartum haemorrhage prevention”, “vaginal birth”, “Caesarean section”, “carbetocin”, “oxytocin”, and “postpartum haemorrhage treatment”. The selection criteria for clinical studies were: (1) Written in English language; (2) vaginal or C-section delivery; and (3) PPH prevention/treatment outcomes were reported. A study was included if the data relevant to the model inputs were available. When multiple reports were available for a model input, the weighted average was adopted as the base-case value and the high/ low values as the range for sensitivity analysis.

**Table 1:** Model input parameters.

Parameters	Base case value	Range for sensitivity analysis	Distribution	Reference
***Clinical inputs***				
Proportion of child births by Caesarean section	0.304	0.243–0.365	Beta	[14]
Probability of PPH ≥500 mL with oxytocin				
Vaginal births	0.022	0.018–0.026	Beta	[6, 8]
Caesarean section	0.110	0.088–0.132	Beta	[6, 8]
Relative risk of PPH ≥500 mL with carbetocin versus oxytocin	0.72	0.56–0.93	Triangular	[8]
Proportion of PPH ≥1500 mL among PPH cases ≥500 mL				
Vaginal births	0.1234	0.0987–0.1481	Beta	[6, 16]
Caesarean section	0.1513	0.1210–0.1816	Beta	[6, 16]
Proportion of emergency hysterectomy among massive postpartum hemorrhage	0.079	0.063–0.095	Beta	[6]
Mortality rate of emergency hysterectomy	0.135	0–0.162	Beta	[17]
***Utility inputs***				
Age (years)	33	18–45	Triangular	[24]
Health utility for females ≥18 years	0.92	-	-	[22]
Disutility				
PPH-related hospitalization without hysterectomy	0.347	0.1–0.7	Triangular	[18]
PPH-related hospitalization with hysterectomy	0.435	0.1–0.7	Triangular	[18]
Length of hospital stay (days)				
Vaginal birth with no PPH	2.38	1.90–2.86	Triangular	[19]
Caesarean section with no PPH	4.52	3.62–5.42	Triangular	[19]
Second-line therapy	6	3–53	Triangular	[16]
Emergency hysterectomy	6.7	4.2–9.2	Triangular	[21]
Increment in length of stay associated with PPH	1.264	1.133–1.395	Triangular	[20]
***Cost inputs*** (USD)				
PPH prevention (per course)				
Oxytocin	1.03–4.10	-	-	Local
Carbetocin	25.6	-	-	Local
PPH treatment (per course)				
Uterotonic agents	63	2–182	Triangular	Local
Second-line therapy	2,765	2,247–3,318	Triangular	Local
Emergency hysterectomy	11,477	10,319–12,634	Triangular	Local
Cost per day				
No PPH	654	-	-	Local
PPH without hysterectomy	654	654–1,750	Triangular	Local
PPH with hysterectomy	654	654–3,128	Triangular	Local

PPH: Postpartum haemorrhage; USD1 = HKD7.8; uterotonic agents for PPH treatment included oxytocin, oxytocin with ergometrine, carboprost.

The proportion of C-section among all child births in the public healthcare sector of Hong Kong (30.4%) adopted the obstetric report of Hospital Authority, under which there were totally eight public obstetric inpatient units serving about 40,000 births per year [14]. The probabilities of PPH ≥500 mL with oxytocin (as PPH prevention intervention) in vaginal birth (0.022) and C-section (0.110) were approximated using (1) the weighted average of PPH ≥500 mL per delivery in the Hong Kong public hospitals between 2013–2016 [6] (prior to the introduction of carbetocin in public hospitals [15], and (2) the relative risk of PPH ≥500 mL of C-section versus vaginal birth derived from the corresponding absolute effect reported by a network meta-analysis of uterotonic agents for PPH prevention (196 clinical trials (n = 135,559) performed in 53 countries) [8]. The probabilities of PPH ≥500 mL with carbetocin (as PPH prevention intervention) at vaginal birth and C-section were estimated using the corresponding probability of PPH ≥500 mL with oxytocin and the relative risk of PPH ≥500 mL with carbetocin (0.72) versus oxytocin [8]. The proportion of cases with blood loss ≥1,500 mL among PPH cases of ≥500 mL in vaginal birth (0.1234) and C-section (0.1513) were estimated from the occurrence of PPH ≥1,500 mL in Hong Kong public hospitals [6] and an outcome analysis of massive primary PPH in Hong Kong [16]. The occurrence of emergency hysterectomy among PPH cases ≥1,500 mL (0.079) were obtained from the statistics of emergency hysterectomy and massive PPH in Hong Kong public hospitals [6]. The mortality of hysterectomy for severe PPH were approximated using the national registry findings on hysterectomy and maternal mortality of patients with severe PPH in Japan [17].

### Disutility inputs

The expected PPH-related QALY loss in each study arm was calculated by the length of hospital stay with a health state and the corresponding disutility. The key PPH-related health states considered for QALY loss estimation were: PPH-related hospitalization without hysterectomy and PPH-related hospitalization with hysterectomy. The disutility values for PPH-related hospitalization with hysterectomy (0.435) and without hysterectomy (0.347) adopted the utility values applied in a cost-effectiveness analysis of PPH management in the US [18]. The length of stay in cases with PPH was estimated by length of stay without PPH in Hong Kong [19] and relative increment of hospital stay with PPH (1.264) versus no PPH [20]. The duration of hospitalization for cases received second-line therapies and hysterectomy were retrieved from clinical outcome studies in Hong Kong [16, 21]. The QALY loss resulted from hysterectomy-related mortality was approximated by the age-specific health utility [22] and age-specific remaining life expectancy (Hong Kong life tables) [23]. The base-case age (33 years) of women who gave birth was retrieved from the Census and Statistics Department of Hong Kong SAR [24]. The mortality-related QALY loss were discounted to current year with an annual rate of 3%.

### Cost inputs

The cost analysis included direct medical costs of major healthcare resource items: Drug acquisition costs of oxytocin and carbetocin for PPH prevention, uterotonic agents for first-line PPH treatment (oxytocin, oxytocin with ergometrine, carboprost), second-line therapy, emergency hysterectomy, and hospitalization.

The public healthcare services provided by the Hospital Authority (the sole public healthcare provider in Hong Kong) are almost completely subsidized by the government. Non-Hong Kong residents are billed upon the charges of healthcare services posted in the Hong Kong Gazette. Assuming the charges listed in the Gazette represent only the cost components (including manpower costs) with no profits, the cost inputs for hospital-related resources (emergency hysterectomy, uterine sutures, cost per day in general ward, high dependency unit and intensive care unit) were therefore approximated using the charges listed in the Hong Kong Gazette. Emergency hysterectomy was the most costly healthcare resource item (USD11,477). Compression suture of uterine was reported as the most common second-line therapy for massive PPH in Hong Kong [16], and the base-case cost input of the second-line therapy therefore adopted the cost of uterine suture (USD2,765). The cost per hospital day adopted the general ward cost (USD654 per day) as the base-case value, and costs for high dependence unit (USD1,750 per day) and intensive care unit (USD3,128 per day) as the upper limit (in sensitivity analysis) for PPH without and with hysterectomy, respectively. All costs were adjusted to year 2022 with an annual discount rate of 3%.

### Cost-effectiveness analysis and sensitivity analysis

The analysis was performed using TreeAge Pro 2022 (TreeAge Software Inc, Williamstown, MA, USA) and Excel 365 (Microsoft Corporation, Redmond, WA, USA). A PPH prevention option was accepted as cost-effective if it resulted in (1) lower QALY loss at lower cost, or (2) lower QALY loss at higher cost and the incremental cost per QALY saved (ICER = ΔCost/ΔQALY loss) was less than the willingness-to-pay (WTP) threshold. In the present model, the gross domestic product per capita of Hong Kong (USD49,630; USD1 = HKD7.8) was adopted as the WTP threshold for highly cost-effective health interventions [25, 26].

The range for sensitivity analysis was either the 95% confidence interval (CI) or high/low values of the model input, if available. If both the 95% CI and high/low values were not reported, ± 20% of the base-case value was used to form the range for sensitivity analysis. Deterministic sensitivity analysis was conducted by variation of each model input over the range for sensitivity analysis specified in Table 1. Probabilistic sensitivity analysis was performed using Monte Carlo simulation. The cost and QALY loss of each study group were recalculated 10,000 times by randomly drawing all model input values simultaneously. The details of distributions used in the probabilistic sensitivity analysis are provided in S1 Table. The difference in cost and QALY loss between carbetocin versus oxytocin of the 10,000 simulations were presented in a scatter plot to determine the probability of carbetocin to be cost-effective.

## Results

### Model validation

The occurrence of clinical events simulated by the present model was validated with the incidence of events reported by the public healthcare provider in Hong Kong. The expected PPH ≥500 mL and emergency hysterectomy in the oxytocin group simulated by the present model were 4.88% and 0.055%, respectively. The model findings were highly similar to the incidence of PPH ≥500 mL (4.8%-5.2%), and emergency hysterectomy (0.04%-0.07%) in 2013–2016 (prior to the introduction of carbetocin in public hospitals) [6].

### Base-case analysis

The expected PPH-related total direct cost, PPH ≥500 mL and ≥1,500 mL, hysterectomy, death, and QALY loss of each study group are shown in Table 2. When compared to the oxytocin group, the carbetocin group reduced PPH-related cost (by USD29 per birth), PPH ≥500 mL and ≥1,500 mL (by 13.7 and 1.9 per 1,000 births), hysterectomy (by 0.15 per 1,000 births), maternal death (by 0.02 per 1,000 births), and saved 0.00059 QALY per birth. The carbetocin was therefore the preferred cost-effective option for PPH prevention of child birth from the perspective of public healthcare provider in Hong Kong.

**Table 2:** Base-case analysis.

PPH Prevention	Total direct cost (USD) (per birth)	PPH ≥500 mL (per 1,000 births)	PPH ≥1,500 mL (per 1,000 births)	Emergency hysterectomy (per 1,000 births)	Death (per 1,000 births)	QALY loss (per birth)
Carbetocin	161	35.1	5.0	0.40	0.05	0.00153
Oxytocin	190	48.8	6.9	0.55	0.07	0.00212

PPH: Postpartum haemorrhage; USD1 = HKD7.8; QALY: quality-adjusted life-year.

### Sensitivity analysis

**Fig 2:**
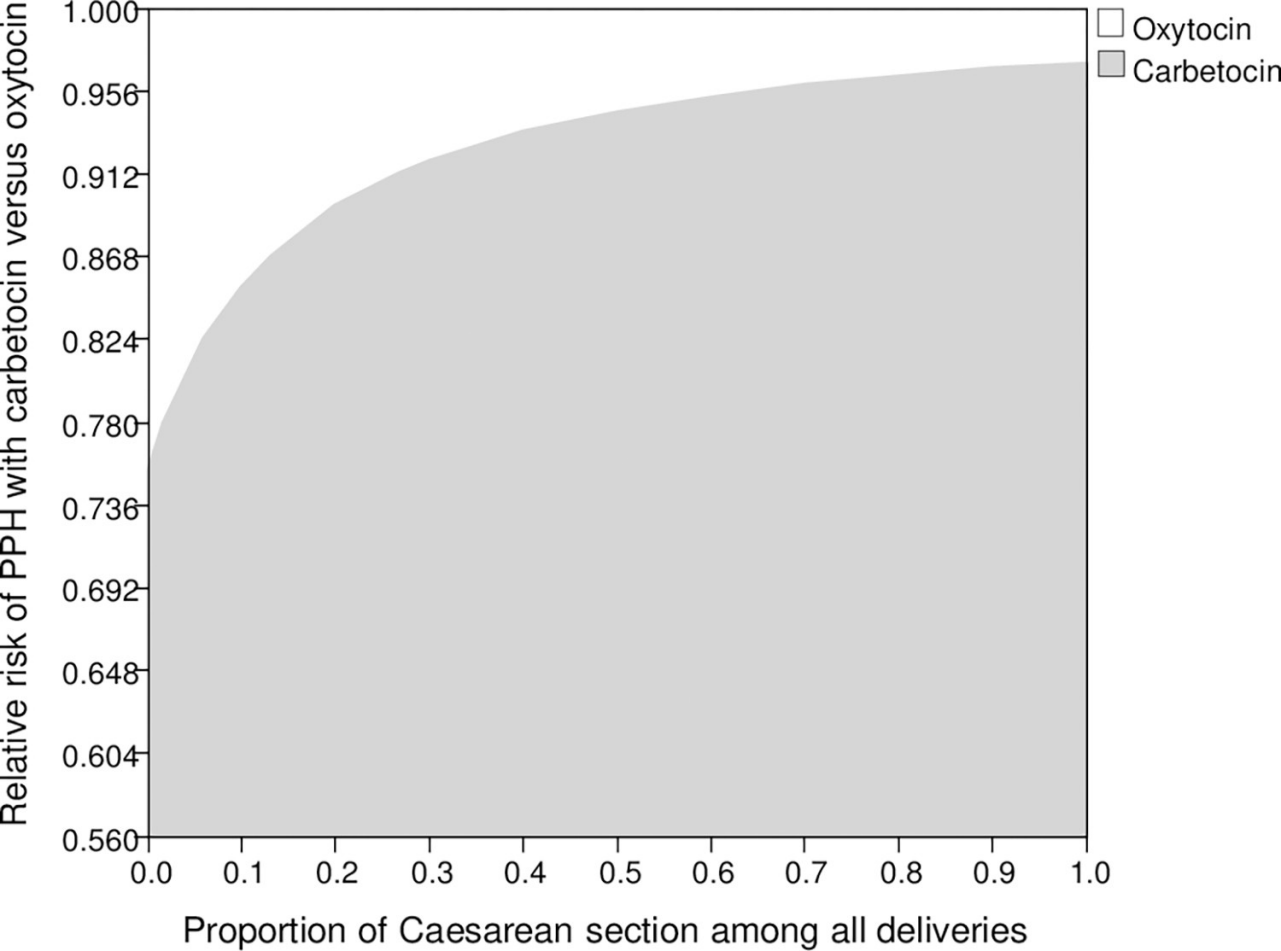
Two-way sensitivity analysis of relative risk of PPH ≥500 mL with carbetocin and proportion of child births by Caesarean section on cost-effectiveness of carbetocin. Threshold line divided the grey zone and white zone; combinations of variables on threshold line had the same cost-effectiveness for carbetocin and oxytocin; white zone: combinations of variables leaded to oxytocin to be cost-effective; grey zone: combinations of variables leaded to carbetocin to be cost-effective. PPH: Postpartum haemorrhage.

In the one-way deterministic sensitivity analysis, the carbetocin group remained to save QALY (comparing to oxytocin) throughout variation of all model inputs (with no threshold value identified). The expected cost-saving generated by the carbetocin group was sensitive to the variation of relative risk of PPH ≥500 mL with carbetocin versus oxytocin (with a threshold value identified: Total cost of carbetocin group was less than that of the oxytocin group when the relative risk of PPH ≥500 mL with carbetocin versus oxytocin was <0.874 (base-case value 0.720). A common influential model input on both the cost and QALY loss was proportion of child births by C-section, despite no threshold value identified. A two-way sensitivity analysis was further performed (with extended ranges) on the relative risk of PPH ≥500 mL with carbetocin and the proportion of child births by C-section. In Fig 2, the white area indicated the combinations of model input values for oxytocin to be cost-effective at the WTP threshold of 49,630 USD/QALY. The grey area indicated the combinations of model input values for carbetocin to be the cost-effective option. At 30% child births by C-section, relative risk of PPH ≥500 mL with carbetocin (versus oxytocin) needed to be <0.919 for carbetocin to be cost-effective. If the proportion of child births by C-section was 100%, relative risk of PPH ≥500 mL with carbetocin needed to be <0.971 for the carbetocin group to be cost-effective. When all child births were vaginal delivery (0% C-section), the carbetocin PPH prevention would be accepted as cost-effective with relative risk of PPH <0.754.

**Fig 3:**
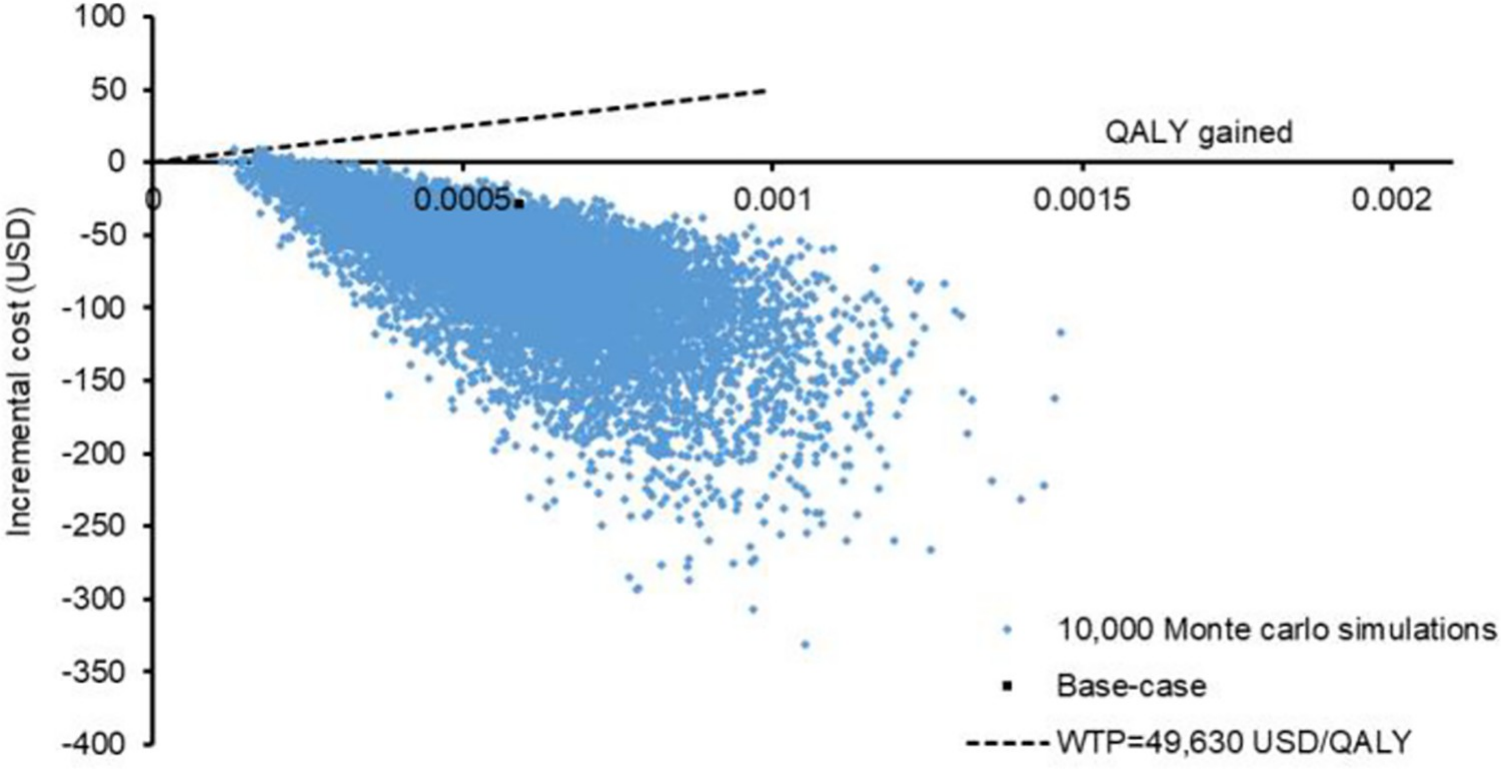
Scatter plot of the incremental cost against QALY saved by the carbetocin group versus the oxytocin group in 10,000 Monte Carlo simulations. QALY: quality-adjusted life-year.

Probabilistic sensitivity analysis was conduction by Monte Carlo simulation of the cost and QALY loss of both groups for 10,000 times. The incremental cost and QALY saved by the carbetocin group versus the oxytocin group are shown in a scatter plot (Fig 3). When comparing to the oxytocin group, the carbetocin group saved cost by USD75 (95%CI USD74 –USD76; *p*<0.001) and saved QALY by 0.000590 (95%CI 0.000586–0.000594; *p*<0.001). The carbetocin group saved cost and QALY in 99.7% and 100% of the 10,000 simulations, respectively, and was accepted as the cost-effective option in >99.7% of time when WTP was lowered to zero USD/QALY.

## Discussion

The present cost-effectiveness analysis examined the outcomes of carbetocin for PPH prevention in women at the third stage of child birth, measured as direct medical cost, PPH, emergency hysterectomy, death and QALY loss in Hong Kong (a low PPH-burden and high-resource city of China). The validity of the model output was demonstrated by the high similarity between the model-simulated and epidemiologic report of the incidence of PPH and emergency hysterectomy. The base-case analysis results found PPH prevention with carbetocin to save QALY (by 0.00059 per patient) and cost (by USD29 per patient) from the perspective of the public healthcare provider in Hong Kong. Despite higher cost per course of PPH prevention with carbetocin versus oxytocin, the total direct medical cost saving and QALY gained by carbetocin were generated from the significant reduction of PPH ≥500 mL (RR 0.72 (95% CI 0.56 to 0.93)) when compared to oxytocin (as demonstrated by the network meta-analysis) [8]. The averted cases of PPH ≥500 mL further reduced healthcare resource utilization of first-line/second-line therapies and emergency hysterectomy for PPH treatment. Beside health economic gain, the reduced need for PPH treatment also reduced the patient-time spent in PPH-related health states with health disutility (thus saved QALYs).

The deterministic sensitivity analysis findings showed the base-case cost-effectiveness results of PPH prevention with carbetocin to be highly robust, with threshold value identified for one influential factor (relative risk of PPH ≥500 mL with carbetocin versus oxytocin). In addition to the effectiveness of carbetocin in preventing PPH, the proportion of child births by C-section was also found to be influential to the variation of both cost and QALY loss in the carbetocin group. As indicated by prior reports, the absolute risk of PPH ≥500 mL was 5-fold higher in C-section births (comparing to vaginal births) [8], and over 70% of delivery mode was C-section in massive PPH cases [16]. The two-way sensitivity analysis on these two influential parameters (relative risk of PPH ≥500 mL with carbetocin and proportion of child births by C-section) over extended ranges found that lower proportion of C-section delivery (higher percentage of vaginal births) required a relatively higher risk reduction of PPH ≥500 mL for carbetocin to be accepted as the cost-effective PPH prevention option. The results of the probabilistic sensitivity analysis further supported carbetocin to be cost-effective in >99.7% of 10,000 Monte Carlo simulations at a WTP of zero USD/QALY.

A systematic review on 15 studies of cost-effectiveness of PPH prevention using uterotonic agents, including 11 studies in low resource settings and three studies in the UK [27]. The three UK studies reported that carbetocin was the preferred cost-effective option for PPH prevention in vaginal births [9, 10] and C-section births [11]. The findings of the systematic review further suggested that the cost-effectiveness findings of uterotonic agents was not generalizable across countries with different resources and health systems [27]. It is therefore important to generate health system and region-specific cost-effectiveness data to inform the decision-maker to optimize the PPH prevention outcomes. The decision-analytic model developed in the present study, including the key clinical, disutility and cost inputs for both vaginal and C-section births, is readily to be adopted in another setting (of different PPH burden and resource level) by using region-specific parameters to inform the model. The model-generated outcomes are also readily to be update with new epidemiology data (when available) to continuously inform the clinicians and healthcare administrators on the optimal use of healthcare resources for PPH prevention.

### Study limitations

There were some limitations in the present cost-effectiveness analysis. Decision-analytical modelling is in general subject to uncertainty of model inputs. The clinical model inputs were extracted from international and local clinical trials. Despite the high transferability of clinical effectiveness findings for cost-effectiveness analyses [28], the clinical inputs sourced from overseas trials might affect the generalizability of model results to patients in Hong Kong. Rigorous sensitivity analyses were therefore performed to examine the impact of model input uncertainty on the robustness of base-case results. A Hong Kong-specific WTP threshold has not yet determined, and a GDP-based WTP threshold was therefore adopted in the present study. We further examined the carbetocin cost-effectiveness over a wide range of WTP threshold, and found carbetocin to save cost and QALY despite the WTP threshold was lowered from 49,630 USD/QALY to zero USD/QALY. The cost analysis included direct medical cost and indirect cost (loss of productivity) was not considered. The model structure was designed over a short timeframe (postpartum hospitalization period), and did not fully take into account the long-term consequence. The long term consequence such as psychological impact associated with serious postpartum events could affect life-long quality of life [29]. The short model time-frame and lack of PPH-associated psychological indexes for QALY assessment might therefore underestimate the economic benefits of PPH prevention with carbetocin.

## Conclusion

PPH prevention with carbetocin at the third stage of child birth (by vaginal or C-section) appeared to save total direct medical cost and QALY from the perspective of public healthcare provider in Hong Kong. The cost-effectiveness acceptance of carbetocin is subject to the risk reduction of PPH associated with carbetocin and the proportion of C-section delivery among all child births.

## Supporting information

S1 Table Probabilistic distributions and parameters used in the probabilistic sensitivity analysis. (DOCX)
