# Supplementary material for: Cost-effectiveness analysis of carbetocin for prevention of postpartum hemorrhage in a low-burden high-resource city of China
Source: PLoS One. 2022 Dec 15;17(12):e0279130. doi: 10.1371/journal.pone.0279130 (PMC9754159; doi:10.1371/journal.pone.0279130)
Supplement: S1 Table — (DOCX) [file pone.0279130.s001.docx]

**Table S1.** Probabilistic distributions and parameters used in the probabilistic sensitivity analysis

| Parameters | **Distribution** |  |  |  |
| --- | --- | --- | --- | --- |
| *Clinical inputs* |  | Alpha parameter | Beta parameter |  |
| Proportion of child births by Caesarean section | Beta | 12,160 | 27,840 |  |
| Probability of PPH ≥500 mL with oxytocin |  |  |  |  |
| Vaginal births | Beta | 612 | 27,228 |  |
| Caesarean section | Beta | 1,338 | 10,822 |  |
|  |  |  |  |  |
| Proportion of PPH ≥1500 mL among PPH cases ≥500 mL |  |  |  |  |
| Vaginal births | Beta | 76 | 536 |  |
| Caesarean section | Beta | 202 | 1,136 |  |
| Proportion of emergency hysterectomy among massive postpartum hemorrhage | Beta | 22 | 256 |  |
| Mortality rate of emergency hysterectomy | Beta | 128 | 819 |  |
|  |  | Minimum | Likeliest | Maximum |
| Relative risk of PPH ≥500 mL with carbetocin versus oxytocin | Triangular | 0.56 | 0.72 | 0.93 |
| *Utility inputs* |  |  |  |  |
| Age (years) | Triangular | 18 | 33 | 45 |
| Disutility |  |  |  |  |
| PPH-related hospitalization without hysterectomy | Triangular | 0.1 | 0.347 | 0.7 |
| PPH-related hospitalization with hysterectomy | Triangular | 0.1 | 0.435 | 0.7 |
| Length of hospital stay (days) |  |  |  |  |
| Vaginal birth with no PPH | Triangular | 1.09 | 2.38 | 2.86 |
| Caesarean section with no PPH | Triangular | 3.62 | 4.52 | 5.42 |
| Second-line therapy | Triangular | 3 | 6 | 53 |
| Emergency hysterectomy | Triangular | 4.2 | 6.7 | 9.2 |
| Increment in length of stay associated with PPH | Triangular | 1.133 | 1.264 | 1.395 |
| *Cost inputs* (USD) |  |  |  |  |
| PPH treatment (per course) |  |  |  |  |
| Uterotonic agents | Triangular | 2 | 63 | 182 |
| Second-line therapy | Triangular | 2,247 | 2,765 | 3,318 |
| Emergency hysterectomy | Triangular | 10,319 | 11,477 | 12,634 |
| Cost per day |  |  |  |  |
| PPH without hysterectomy | Triangular | 654 | 654 | 1,750 |
| PPH with hysterectomy | Triangular | 654 | 654 | 3,128 |
